# “A traumatic path to the profession” obstetric violence through the eyes of student nurses: a qualitative study

**DOI:** 10.1590/1980-220X-REEUSP-2025-0075en

**Published:** 2026-02-27

**Authors:** Pelin Calpbinici, Aynur Kizilirmak, Nur Şifa Yavuz

**Affiliations:** 1 Nevşehir Hacı Bektaş Veli University, Semra and Vefa Küçük Faculty of Health Sciences, Department of Obstetrics and Gynecology Nursing, Nevsehir, Turkey.

**Keywords:** Obstetric Violence, Students, Nursing, Qualitative Research, Labor, Obstetric, Violência Obstétrica, Estudantes de Enfermagem, Pesquisa Qualitativa, Trabalho de Parto

## Abstract

**Objective:**

To explore student nurses’ experiences and perceptions of obstetric violence.

**Methods:**

A qualitative exploratory design was adopted. Fourteen nursing students who completed the obstetrics and gynecology nursing course and clinical practice were selected through purposive sampling. Data were collected using in-depth, semi-structured interviews.

**Results:**

Thematic analysis revealed two conceptual levels. Micro-level themes included: Lack of Information and Consent, Verbal Harassment, Loss of Professional Value (with subthemes Barrier to Education and Negative Role Modelling), and Lack of Effective Communication (with subthemes Language Barriers and Use of Vulgar Language). The macro-level theme, Seeking for Solutions, comprised the subthemes Awareness and Empathizing. Students emphasized the emotional and ethical challenges they faced when witnessing disrespectful maternity care, which often conflicted with their formal training.

**Conclusions:**

Witnessing obstetric violence had a profound psychological and educational impact on student nurses, often experienced as traumatic. Findings highlight the need to address obstetric violence in nursing education to strengthen awareness, empathy, and commitment to respectful, womancentered maternity care.

## RESUMO

**Objetivo:**

Explorar as experiências e percepções de estudantes de enfermagem sobre a violência obstétrica.

**Métodos:**

Abordagem qualitativa exploratória. Quatorze estudantes de enfermagem que concluíram o curso de enfermagem em obstetrícia e ginecologia e o estágio clínico foram selecionadas por amostragem intencional. Os dados foram coletados por meio de entrevistas semiestruturadas em profundidade.

**Resultados:**

A análise temática revelou dois níveis conceituais. Os temas de nível micro incluíram: Falta de Informação e Consentimento, Assédio Verbal, Perda de Valor Profissional (com os subtemas Barreira à Educação e Modelagem Negativa) e Falta de Comunicação Eficaz (com os subtemas Barreiras Linguísticas e Uso de Linguagem Vulgar). O tema de nível macro Busca por Soluções compreendeu os subtemas Conscientização e Empatia. Os estudantes enfatizaram os desafios emocionais e éticos que enfrentaram ao presenciar cuidados maternos desrespeitosos, que frequentemente entravam em conflito com sua formação acadêmica.

**Conclusões:**

Presenciar violência obstétrica teve um profundo impacto psicológico e educacional nos estudantes de enfermagem, que é frequentemente vivenciado como traumático. Os resultados destacam a necessidade de abordar a violência obstétrica na formação em enfermagem para fortalecer a conscientização, a empatia e o compromisso com o cuidado materno respeitoso e centrado na mulher.

## INTRODUCTION

Obstetric violence is recognized as a widespread problem on a global scale^(1,2)^. In a study conducted by Castro and Frías^(2)^ among Mexican women aged 15–49 years, 33.3% of women reported experiencing obstetric violence in their most recent birth in the preceding five years. Such negative experiences have been noted to adversely affect women’s mental health in the postpartum period, possibly leading to postpartum depression^(3)^, exacerbating postpartum sexual dysfunction and feelings of anger and distrust^(4)^. Additionally, due to obstetric violence, women may avoid future pregnancies and thus experience secondary infertility^(4)^, and they may have an increased risk of developing post-traumatic stress disorder one year after birth^(5)^.

In a “constructivist grounded theory” study conducted in Spain among healthcare professionals and students, Mena-Tudela et al.^(4)^ revealed that obstetric violence impacts not only the woman receiving care but also future healthcare professionals (students) who witness these processes. According to this approach, obstetric violence is a multi-layered phenomenon shaped by numerous interacting factors such as structural problems in the health system, perceptions of gender, professional hierarchies, and normalized practices in clinical settings. Within this context, students and professionals sometimes characterize practices that generate violence as “routine” or “unavoidable,” which contributes to the invisibility of violence^(4)^. Indeed, the normalization practices contributing to obstetric violence makes it difficult for healthcare professionals to recognize these actions as abuse/violence^(6,7)^. Therefore, increasing the awareness of obstetric violence among student nurses, who are the future healthcare professionals, is an important step toward its prevention. Moreover, highlighting the mental and emotional burden stemming from the examples of obstetric violence that students either witness or personally experience during clinical practice could enhance the sensitivity and capacity for action among healthcare workers and educators. Accordingly, the first step in preventing obstetric violence is ensuring both current and future caregivers (students) multidimensionally understand and define the concept. The study aimed to examine student nurses’s experiences and perceptions of obstetric violence using a qualitative exploratory design.

## METHOD

### Study Design

A qualitative exploratory design was adopted with a focus on understanding student nurses’ experiences and perceptions of obstetric violence. Given the aim of exploring how participants made sense of their clinical observations, a reflexive thematic analysis approach was used in data analysis. This method was chosen for its flexibility and suitability in capturing both the explicit and implicit meanings within participant narratives. This study was reported using the Consolidated Criteria for Reporting Qualitative Research (COREQ) guidelines^(8)^.

### Population and Sample of the Study

Participants were undergraduate nursing students from a faculty of health sciences in Türkiye who had completed the obstetrics and gynecology nursing course and clinical practice in labor wards. A purposive maximum variation sampling strategy was employed to ensure diversity in age, gender, year of study, and clinical exposure.

The participant inclusion criteria were as follows: (1) volunteering to participate in the study, (2) being a student nurse, (3) having taken the obstetrics and gynecology nursing course, (4) knowing the concept of obstetric violence, and (5) experiencing obstetric violence.

### Sample Size

A total of 14 students participated in the study. Sample size was determined based on the principle of data saturation, which was reached when no new themes or insights emerged during the interviews. After the 12^th^ interview, recurring patterns were identified, and two additional interviews confirmed thematic redundancy.

### Data Collection Tools

The data collection tools consisted of a personal information form containing introductory information about the participants and a semi-structured interview form.

***Personal information form:*** The form consisted of a total of four questions about students’ age, gender, year of education, and marital status.

***Semi-structured interview form:*** This form was developed in accordance with the research purpose and relevant literature^(9)^. The study aimed to evaluate the feelings, thoughts and observations of student nurses regarding obstetric violence. Based on the constructivist grounded theory and research purpose, a semi-structured interview form consisting of seven open-ended questions was created (Table 1).

**Table 1:** Semi-structured interview form – Nevsehir, Türkiye, 2023.

1	What does the concept of Obstetric Violence remind you of?
2	What characteristics would you include in your definition of obstetric violence?
3	How did you feel when you witnessed obstetric violence?
4	What beliefs, values, or principles do you think are associated with obstetric violence and why?
5	What consequences do you think obstetric violence has on patients (physically, psychologically and sexually)?
6	What strategies do you believe are necessary to avoid obstetric violence (at the individual, health, and institutional levels)?
7	Is there anything else you’d like to add?

### Data Collection

The data were collected by the researchers between January and February 2023 using the aforementioned tools. The semi-structured interview guide was used to ensure consistent questions for all participants, with the specific questions listed in Table 1.

Students (60) who received obstetrics and gynecology nursing and completed the delivery room practice internship were informed about the research process and invited to participate in the study. Twenty students voluntarily applied to the study. The students were invited for interviews on specific days and times set by the researchers. Interviews were conducted until reaching data saturation. When the concepts and expressions answering the study questions began to repeat (i.e., when saturation was reached), data collection was terminated. In this study, the interviews with 14 participants were audio recorded with use of a voice recorder. The data collection was conducted in a suitable environment (the researchers’ study room). Participants and researchers were positioned so that they could see each other during the interview. The first researcher conducted the interview while the second researcher took observation notes. The questions were asked in the same order and additional explanations were given when necessary. The researcher asked questions such as “Can you give more details?”, “Can you elaborate on this?”, “What do you think about this subject?”. All opinions expressed were evaluated as qualitative data. The interviews lasted 30–35 minutes on average.

### Data Analysis

The data were analyzed using van Manen’s thematic analysis^(10)^. A three-stage textual analysis was conducted, consisting of (1) naive reading, (2) structural analysis, and (3) a comprehensive understanding that included discussion^(10)^. In the naive reading stage, the scope and meanings of the text were estimated naively without any formal analysis. At this stage, the interview text was read several times to gain a general impression. In the structural analysis stage, initial codes were created to uncover the meanings, and the first codes were categorized into themes. The themes were generated inductively by the researchers in accordance with reflexive thematic analysis. Coding was carried out separately by two researchers, and any inconsistencies between the coders were discussed until a consensus was reached. Before determining the analytical themes, the most prominent codes, their descriptions, and the relationships among them were identified and then combined into descriptive themes using thematic synthesis. In the final stage, the data obtained from the previous two stages and the objectives of the study were reviewed as a whole in the light of the literature.

### Reliability and Validity of the Study

The validity and reliability of this study were ensured through rigor, consistency, transferability, and confirmability. Before the interview, the necessary information was provided to ensure safe communication between the participant and the researcher. With the participant’s consent, one researcher conducted the interviews while the other observed. During the interviews, the researchers avoided leading questions, and at the end of the interview, the participant was asked if he/she had anything to add and additional thoughts and comments were recorded. Interview transcripts were shared with the participants at the end of the interview and informed consent was obtained. The validity of the study was supported by frequently including direct statements of the participants in the manuscript. For confirmability, interview notes (voice recordings) were used as raw data or notes were taken on the statements of the participants during the interview, and their statements were directly included in the research report. In addition, the study design (research questions, data collection, and data evaluation) was explained in detail to ensure consistency. Thus, it was ensured that readers would understand the method and the study. The researchers believe the interview results from this sample group are transferable and consistent, as they can be applied to similar sample groups in different settings.

### Self-Reflexive Knowledge of the Researchers

The self-reflexive knowledge of the researchers in this study is as follows: the first and second researchers completed their doctorates (PhD) in the field of obstetrics and gynecological nursing. The researchers have scientific research experience in gynecology and qualitative research. Moreover, the first author has given birth once, an experience that, to a certain extent, facilitated her understanding of other women’s experiences. In addition, the first author had a high-risk pregnancy and spent 52 days in the gynecology ward for preterm delivery risk and received medical care. Furthermore, the researcher’s background as a gynecology nurse ensured both the effective communication with the participants and the consistency of the interview process.

### Ethical Approval

Written approval was obtained from the Ethics Committee of the relevant University before starting the research (Date: 21.11.2022, Issue No.: 2022.10.108). Written permission was obtained from the relevant institution to conduct the research (Date: 09.12.2022, Issue No.: 51301242-200-2200001428). The study was conducted in accordance with the principles in the Declaration of Helsinki, and written informed consent was obtained from each participant by explaining the purpose of the study and stating that a voice recorder would be used during the interview. The names of the participants were kept confidential and coded as P1, P2,…, P14 (instead of full names).

## RESULTS

Of the students, 71.4% (n = 10) were female and 28.6% (n = 4) were male. Their ages ranged between 21 and 31 years (21.50 ± 2.79). Thematic analysis produced two conceptual strata in line with the micro/macro level framework proposed by Mena-Tudela et al.^(9)^: **Micro-level themes** included: (1) *Lack of Information and Consent*, (2) *Verbal Harassment*, and (3) *Loss of Professional Value*, which encompassed the subthemes Barrier to Education and Negative Role Modelling. (4) *Lack of Effective Communication* which comprised the subthemes Language Barriers and Use of Vulgar Language. The **macro-level theme** was *Seeking for Solutions* and incorporated the subthemes Awareness and Empathizing (Table 2).

**Table 2:** Themes and subthemes emerging from the Interviews – Nevsehir, Türkiye, 2023.

Conceptual model level		Themes	Subthemes	Participant statements
Micro-level strategies	Non-consented care	Lack of information and consent		Students stated that the information provided to women during childbirth was incomplete and inadequate, and in some cases, women were not given any information at all.
	Verbal abuse and infantilization of women	Verbal Harassment		Students reported that midwives yelled at women, insulted them, made negative comments, and used derogatory expressions.
	Relationship hierarchy in the healthcare system	Loss of professional value	A barrier to education	Students stated that observing obstetric violence negatively affected their education and this situation reduced their desire to learn.
			Negative role model	Students stated that health personnel were negative role models because of their attitudes toward patients and thought that this situation would harm the development of their competencies.
	Pregnancy and childbirth Intervention and unnecessary medicalization	Lack of effective communication	Language barriers	The communication problem damaged the relationship between the midwife and the woman, paving the way for negative practices against women and preparing the ground for interventions that should not be done
			Use of vulgar Language	Students stated that the rude language used by midwives while communicating with women prevented them from receiving health services.
Macro-level strategies	Institutional, organizational and political interventions	Seeking for Solutions	Awareness	Students stated that in-service training should be provided to increase the awareness of health professionals on providing humane care.
			Empathizing	Students stated that health workers should not normalize obstetric violence and should empathize with the person or a family member who may be in the same environment. Some students stated that regular trainings should be provided to improve empathy skills in healthcare professionals.

### Micro-Level Strategies

#### Theme 1. Lack of Information and Consent

Students stated that the information provided to women during childbirth was incomplete and inadequate. In some cases, women were not given any information at all, as although they requested clarification about the decisions taken and practices, they were not informed. Women’s consent was also ignored during the procedures. Students reported that midwives did not engage in much dialog with women, particularly foreign national women (mostly Syrian refugees), and ignored most of their decisions. According to many students, most pregnant women who came to the delivery room were anxious because they did not know the process, and midwives did not provide any explanation about the process. They stated that the midwives did not consider the woman’s decision and a woman was rushed into a cesarean section without any explanation.

*“I witnessed that they did not provide any information about when the patient would be discharged and how the treatment would proceed.*” (P8)

*“The woman’s baby was taken to the neonatal intensive care unit after birth. However, neither the doctor nor the nurse informed the patient’s relatives about the baby’s condition.*” (P5)

*“Nurses provided care and treatment to patients in the ward, but they did not provide any information or explanation to the patients.*” (P8)

#### Theme 2. Verbal Harassment

Students reported that midwives yelled at women, insulted them, made negative comments, and used derogatory expressions. Sometimes this treatment of women was also related to their decision to conceive or their future ability to give birth. Midwives sometimes threatened women with a lack of assistance during delivery if they did not push effectively.

These experiences deeply contrasted with the ideal models of therapeutic communication taught in nursing education.

*“In reality, what we are taught and the actual delivery room environment are very different. We were supposed to provide psychological support to the woman. Probably our psychological support would have reduced her pain. However, midwives behaved in the opposite way, in a way that would increase the woman’s pain or traumatize her*.” (P12)

*“I remember a midwife yelling at a woman, ‘You have been coming here for four days, you have been bothering us, are you not supposed to give birth? Give birth already, enough is enough*’.” (P3)

*“The woman was pushing, she was in pain, and the midwives and nurses were constantly yelling, mocking her, and saying insulting things like ‘How did you give birth before? How will you give birth? Did you really give birth to two children? Do not give birth again*’.” (P7)

#### Theme 3: Lack of Effective Communication

##### Subtheme 1: Language Barriers

Students stated that the language barrier led to communication difficulties between foreign women and midwives. The majority of foreign women were of Syrian nationality, and their mother tongue was Arabic. The fact that none of the midwives spoke Arabic was a major obstacle to effective communication. Thus, this communication problem damaged the relationship between the midwife and the woman, paving the way for negative practices against women and preparing the ground for interventions that should not be done.

“*Foreign nationals give birth a lot and have language problems, and I think healthcare workers have difficulty in providing care because they do not understand the spoken language*.” (P7)

Such communication breakdowns affect the physical and emotional state of women in labor.

“*Women go hungry for long periods of time during childbirth and midwives are unable to explain the reasons for this to foreign women. This affects women’s ability to concentrate on childbirth*.” (P6)

“*The midwives did vaginal examinations all the time. Some women even closed their legs and refused to have it done*.” (P1)

##### Subtheme 2: Use of Vulgar Language

Students emphasized that they were disturbed by the approach of midwives when communicating with women. Midwives often approached women with impatience and lack of understanding by using imperatives such as “do this, do that”, which can cause women to withdraw and not receive the necessary healthcare.

This tone of command further distanced patients from asking questions or understanding their care.

“*When the patient asks something, they often speak harshly, which causes the patient to withdraw even more and not be able to ask about the practices they are curious about and want to ask about.*” (P2)

#### Theme 4: Loss Of Professional Value

Students reported that the obstetric violence they observed negatively affected them and that this situation was a negative example for their profession and constituted an obstacle to their education.

##### Subtheme 1: Barrier to Education

Students stated that observing obstetric violence negatively affected their education and this situation reduced their desire to learn.

Exposure to violence in clinical settings distracted students from educational objectives.

“*I focused more on the violence when I should be watching the vaginal delivery explained in class.*” (P2)

“*It was the first time I had seen childbirth. I thought it was something very painful. I thought it was humiliating... It was chaos for me, and I felt more stressed*.” (P7)

Students expressed difficulty reconciling theoretical knowledge with clinical reality.

“*This is not how we were told; the moment we try to do it the way we were told, we feel like we cannot communicate effectively with the patient.*” (P10)

##### Subtheme 2: Negative Role Model

Students stated that health personnel were negative role models because of their attitudes toward patients and thought that this situation would harm the development of their competencies. Indeed, students observe their mentors, accept them as role models, and learn from them. This role modeling may cause them to internalize professional values in a negative way. It is crucial to prepare the next generation of nurses for a professional working environment. Negative attitudes toward women can threaten students’ progress and motivation.

“*In our four-year undergraduate education, we’ve always learned about therapeutic communication methods and being understanding toward patients. Witnessing such negative attitudes in the hospital while learning these things makes me worry if I will be like that in the future*.” (P13)

Another student stated clearly how this misalignment between theory and practice led to emotional withdrawal:

“*It was not what I learned. It really demotivated me*.” (P12)

The emotional toll of witnessing obstetric violence extended beyond demotivation. Some students felt deeply traumatized, isolated and abandoned, expressing experiences of profound helplessness:

“*You are on your own, you are going to give birth, and you are very lonely. The closest person a patient can hold on to in a hospital is a healthcare worker, but you do not get the support. It was a very traumatic event for me, and I was greatly affected*.” (P3)

Another participant described a similar emotional impact from witnessing disrespectful treatment:

“*It was the first time I had seen childbirth. I thought it was something very painful. I thought it was humiliating, and I was very upset. A solution must be found for this situation, and if necessary, appropriate strategies must be developed. If the reason for this is a lack of personnel, the number of personnel must be increased*.” (P7)

Overall, these narratives demonstrate that negative role modeling by health personnel can profoundly impact students, eliciting significant emotional distress, feelings of hopelessness, and potentially compromising their motivation, ethical orientation, and professional preparedness. Ensuring students have ethically grounded and compassionate mentors is essential to fostering future healthcare providers committed to respectful, empathetic, and humane care.

### Macro-Level Strategies

#### Theme 5. Seeking for Solutions

The students stated that the solution to obstetric violence is to increase the awareness of health personnel and empathize with them.

##### Subtheme 1: Awareness

Students recommended that in-service training for healthcare professionals should focus on improving effective communication and providing detailed information about the childbirth process and procedures to support women during childbirth, thereby increasing awareness of the need to provide humane and supportive care to women. Most students stated that information about obstetric violence should be given in their undergraduate education to raise awareness among their peers.

“*I think nurses can be trained regarding empathy, and their empathy skills can be improved*.” (P8)

Some also advocated for early integration of obstetric violence topics in formal nursing education.

“*Our lecturers constantly talk about what we should do from A to Z, and how we should approach the patient. Providing information about obstetric violence in our classes will increase our awareness on this issue. This will prevent us from unknowingly harming the patient in the future. I think a comprehensive training is necessary.*” (P2)

##### Subtheme 2: Empathizing

The students stated that health workers should not normalize obstetric violence and should empathize with the person or a family member who may be in the same environment. Some students stated that regular trainings should be provided to improve empathy skills in healthcare professionals. These remarks underscored the importance of reflective practice and humanistic values in clinical care.

“*They should provide care by asking themselves, If I were in this situation, how would I want to be treated? And should never normalize violence.*” (P14)

“*Healthcare workers should never forget that the individual in front of them is a human being*.” (P12)

“*A conscientious approach and empathy are very important. As women, we can all be in this situation at any time. I don’t do something to the other person that I don’t want to be done to me, or I don’t do something that will lead to a complaint; I think this is not ethical behavior at all. In addition, in order to improve the empathy skills of health workers, regular trainings should be provided and they should be reminded of this situation.*” (P6)

## DISCUSSION

In this study, nursing students’ experiences related to obstetric violence were examined using a qualitative exploratory design and reflexive thematic analysis. The themes that emerged from the study indicate psychological and verbal violence in the student nurses’ narratives and observations. Students reported that women were frequently exposed to forms of verbal violence such as insults, yelling, threats, and scolding during labor and delivery. Similarly, many studies found that women face negative, disrespectful, and dishonorable attitudes during labor^(11,12)^. One study reported that women were exposed to negative behaviors including physical abuse such as slapping and stigmatization, discrimination such as locking them in the toilet, and verbal abuse such as yelling and intimidation^(12)^. In a study examining the childbirth experiences of women who gave birth in health facilities in low- and middle-income countries, women were frequently subjected to negative birth experiences, including neglect, abandonment, denial of pain relief, lack of informed consent, and painful vaginal examinations^(11)^. In some cases, health workers may be unaware of obstetric violence and abuse their professional authority to undermine women’s rights, freedom, decisionmaking capacity, and ultimately, their autonomy^(13)^. Indeed, in the present study, students reported that women were not included in decisions and interventions and healthcare workers often did not provide any explanations. Similarly, another study reported that medical interventions were made without the consent/permission of women during labor and delivery, and that they were misled or not informed sufficiently^(14)^. According to the European Charter of Patients’ Rights^(15)^. “Every individual has the right of access to information about his or her state of health, health services and how to use them, and information generated by scientific research and technological innovation.” Therefore, every midwife/nurse should inform women before each procedure, considering the women’s autonomy.

Culturally competent nursing is defined as care that is sensitive to different cultural characteristics – such as language, communication, beliefs, and behavior – which can ultimately improve healthcare outcomes^(16)^. Among these features, communication is of particular importance because the existence of a common language is a basic requirement for maintaining care and evaluating its effectiveness^(17)^. Many studies have shown that communication and language barriers are among the most common problems in providing care to foreign patients^(17,18)^. An important observation of student nurses who participated in the present study was language barrier. According to them, there was a communication problem between foreign women and midwives because of language barriers, and this paved the way for negative practices toward women. To overcome this situation, having a sufficient number of interpreters to assist nurses in communicating with foreign patients in health institutions would be an important step to ensure dignified care during labor and delivery.

Violence in maternity care toward women can lead to distrust of the health system and existing supportive structures. One study showed that women described the hospital and healthcare professionals as a hateful environment^(19)^. Another study reported that mothers’ distrust in the healthcare system because of obstetric violence is a strong deterrent and negatively affects their future health-seeking behaviors^(20)^. The most important duties of nurses are to eliminate this situation, ensure that women receive the highest possible quality of care, and provide services that uphold human dignity. In the present study, students suggested increasing the awareness of nurses as a solution to obstetric violence. Another study showed the possibility to change this perception with the implementation of education on obstetric violence^(21)^. They argued that policies, guidelines, protocols, and education, as well as training that promotes respect, informs and raises awareness among health professionals, will eliminate obstetric violence. Moreover, providing information about childbirth to every woman in the prenatal period may improve the experience of childbirth.

In addition to the negative impact of obstetric violence on women’s lives, witnesses may experience secondary traumatic stress or compassion fatigue^(22)^. The results obtained in the present study are consistent with the literature, and students reported that witnessing obstetric violence made them feel bad. On the other hand, clinical practice in nursing education constitutes an essential component of the educational process, allowing students the opportunity to apply theoretical knowledge into practical situations^(23)^. The student can be influenced, either positively or negatively, by the skills, knowledge, and attitudes demonstrated by professional nurses in the clinical setting. Positive role modeling significantly enhances the caring skills of student nurses in clinical practice. In particular, the nursing skills of student nurses are influenced by the humane behavior and respectful communication skills they observe in their role models^(24)^. Witnessing inappropriate practices and having negative role models adversely affects the development of professional identity among student nurses^(25)^. Indeed, in this study, student nurses reported that the midwives who engaged in obstetric violence were perceived as a negative role model, impacting their education negatively and diminishing their desire to learn. Similar to these findings, previous research indicates that students’ exposure to negative role models in the clinical setting adversely affected their learning and resulted in negative feelings toward the nursing profession^(26)^. Furthermore, the exposure of student nurses to unethical behaviors exhibited by professional nurses toward patients may lead them to normalize and adopt such behaviors. Student nurses, who are at the very beginning of their profession, must have competent role models who prioritize the long-term health of society. This ensures that they can deliver effective and successful patient care while also providing dignified maternal care in obstetric clinics. Furthermore, incorporating obstetric violence into nursing curricula can enhance the awareness of nurses on this issue and may be an important step toward preventing obstetric violence.

## LIMITATIONS

Despite the efforts to maximize sample diversity, the findings of this qualitative study – due to its inherent nature – cannot be generalized to all student nurses in Turkey. The findings obtained in the present study are limited to the analysis of qualitative data and the cultural environment and socioeconomic status of the student nurses participating in the study. Although the results cannot be generalized, the findings of the study are instructive for other research, as they present important details and categories as a conceptual model and illustrate exemplary experiences.

## IMPACT ON PRACTICE AND RESEARCH

To improve the quality of maternal health services, nurses must take an active role in creating laws that prevent obstetric violence, a clear violation of women’s human rights. To eliminate the maltreatment of women in maternal health services, legal regulations must be coupled with training for health professionals on ethics, gender equality and human rights, raising their awareness on these critical issues. To ensure positive transformation in maternal health services, research should be conducted using various methodologies to explore the causes, prevalence and consequences of obstetric violence. Simultaneously, effective training programs should be developed.

## CONCLUSION

Student nurses who witnessed obstetric violence during their education were negatively affected by this situation and this event was traumatic for them. Mistreatment of women during labor is a problem related to the quality of care and a violation of human rights. Every woman, no matter where she is in the world, has the right to receive dignified healthcare free from all forms of abuse. Nurses have a key role in ensuring that women receive quality maternity care during pregnancy, childbirth, and the postpartum period. They are in an ideal position to advocate for and protect women, serving as the primary line of defense against abusive and disrespectful care. Finally, it is considered that the theme has its merits, and that the understanding and early awareness of students—almost nursing professionals—toward practices that neglect good care and violate the human rights and autonomy of pregnant women should be valued. Therefore, the findings of this study are valuable for developing intervention strategies to address obstetric violence experienced by women and for delivering quality, egalitarian, and personalized care.

## Data Availability

The data that support the findings of this study are available from the corresponding author, upon reasonable request.
