# Supplementary material for: “A traumatic path to the profession” obstetric violence through the eyes of student nurses: a qualitative study
Source: Rev Esc Enferm USP. 2026 Feb 27;60:e20250075. doi: 10.1590/1980-220X-REEUSP-2025-0075en (PMC12965747; doi:10.1590/1980-220X-REEUSP-2025-0075en)
Supplement: Translation

# Translation: “Un camino traumático hacia la profesión” la violencia obstétrica desde la perspectiva de estudiantes de enfermería: un estudio cualitativo

**DOI:** 10.1590/1980-220X-REEUSP-2025-0075es

**Authors:** Pelin Calpbinici, Aynur Kizilirmak, Nur Şifa Yavuz

**Affiliations:** 1 Nevşehir Hacı Bektaş Veli University, Semra and Vefa Küçük Faculty of Health Sciences, Department of Obstetrics and Gynecology Nursing, Nevsehir, Turkey.

**Keywords:** Violencia Obstétrica, Estudiantes de Enfermería, Investigación Cualitativa, Trabajo de Parto

## Abstract

**Objetivo:**

Explorar las experiencias y percepciones de los estudiantes de enfermería sobre la violencia obstétrica.

**Métodos:**

Se adoptó un diseño exploratorio cualitativo. Se seleccionó mediante muestreo intencional a catorce estudiantes de enfermería que completaron el curso de enfermería de obstetricia y ginecología y la práctica clínica. Los datos se recopilaron mediante entrevistas semiestructuradas en profundidad.

**Resultados:**

El análisis temático reveló dos niveles conceptuales. Los temas a nivel micro incluyeron: Falta de Información y Consentimiento, Acoso Verbal, Pérdida de Valor Profesional (con los subtemas Barreras a la Educación y Modelos de Rol Negativo) y Falta de Comunicación Efectiva (con los subtemas Barreras Lingüísticas y Uso de Lenguaje Vulgar). El tema a nivel macro, Búsqueda de Soluciones, comprendió los subtemas Conciencia y Empatía. Los estudiantes enfatizaron los desafíos emocionales y éticos que enfrentaron al presenciar una atención materna irrespetuosa, que a menudo entraba en conflicto con su formación académica.

**Conclusiones:**

Ser testigo de violencia obstétrica tuvo un profundo impacto psicológico y educativo en los estudiantes de enfermería, que a menudo se experimentó como traumático. Los resultados resaltan la necesidad de abordar la violencia obstétrica en la formación de enfermería para fortalecer la concienciación, la empatía y el compromiso con una atención materna respetuosa y centrada en la mujer.

## INTRODUCCIÓN

La violencia obstétrica se reconoce como un problema generalizado a escala mundial^(1,2)^. En un estudio realizado por Castro y Frías^(2)^ entre mujeres mexicanas de entre 15 y 49 años, el 33,3% de las mujeres declararon haber sufrido violencia obstétrica en su último parto en los cinco años anteriores. Se ha observado que estas experiencias negativas afectan negativamente a la salud mental de las mujeres en el posparto, lo que puede provocar depresión posparto^(3)^, exacerbar la disfunción sexual posparto y los sentimientos de ira y desconfianza^(4)^. Además, debido a la violencia obstétrica, las mujeres pueden evitar futuros embarazos y, por lo tanto, experimentar infertilidad secundaria^(4)^, y pueden tener un mayor riesgo de desarrollar un trastorno de estrés postraumático un año después del parto^(5)^.

En un estudio de “teoría fundamentada constructivista” realizado en España entre profesionales de salud y estudiantes, Mena-Tudela et al.^(4)^ revelaron que la violencia obstétrica afecta no solo a la mujer que recibe la atención, sino también a los futuros profesionales de salud (estudiantes) que son testigos de estos procesos. Según este enfoque, la violencia obstétrica es un fenómeno multifacético moldeado por numerosos factores que interactúan entre sí, como los problemas estructurales del sistema sanitario, las percepciones de género, las jerarquías profesionales y las prácticas normalizadas en los entornos clínicos. En este contexto, los estudiantes y los profesionales a veces caracterizan las prácticas que generan violencia como “rutinarias” o “inevitables”, lo que contribuye a la invisibilidad de la violencia^(4)^. De hecho, las prácticas de normalización que contribuyen a la violencia obstétrica dificultan que los profesionales de la salud reconozcan estas acciones como abuso/ violencia^(6,7)^. Por lo tanto, aumentar la conciencia sobre la violencia obstétrica entre los estudiantes de enfermería, que son los futuros profesionales de la salud, es un paso importante hacia su prevención. Además, destacar la carga mental y emocional derivada de los ejemplos de violencia obstétrica que los estudiantes presencian o experimentan personalmente durante la práctica clínica podría mejorar la sensibilidad y la capacidad de acción de los trabajadores de salud y los educadores. En consecuencia, el primer paso para prevenir la violencia obstétrica es garantizar que tanto los cuidadores actuales como los futuros (estudiantes) comprendan y definan el concepto de forma multidimensional. El estudio tenía como objetivo examinar las experiencias y percepciones de los estudiantes de enfermería sobre la violencia obstétrica utilizando un diseño exploratorio cualitativo.

## MÉTODO

### Diseño del Estudio

Se adoptó un diseño exploratorio cualitativo centrado en comprender las experiencias y percepciones de los estudiantes de enfermería sobre la violencia obstétrica. Dado el objetivo de explorar cómo los participantes interpretaban sus observaciones clínicas, se utilizó un enfoque de análisis temático reflexivo en el análisis de los datos. Se eligió este método por su flexibilidad y adecuación para captar tanto los significados explícitos como implícitos de las narrativas de los participantes. Este estudio se informó utilizando las directrices de los Criterios Consolidados para Informar sobre Investigación Cualitativa (COREQ)^(8)^.

### Población y Muestra del Estudio

Los participantes eran estudiantes de enfermería de una facultad de ciencias de la salud de Turquía que habían completado el curso de enfermería obstétrica y ginecológica y la práctica clínica en salas de parto. Se empleó una estrategia de muestreo intencional de máxima variación para garantizar la diversidad en cuanto a edad, género, año de estudio y exposición clínica.

Los criterios de inclusión de los participantes fueron los siguientes: (1) participar voluntariamente en el estudio, (2) ser estudiante de enfermería, (3) haber cursado la asignatura de enfermería obstétrica y ginecológica, (4) conocer el concepto de violencia obstétrica y (5) haber sufrido violencia obstétrica.

### Tamaño de la Muestra

Un total de 14 estudiantes participaron en el estudio. El tamaño de la muestra se determinó basándose en el principio de saturación de datos, que se alcanzó cuando no surgieron nuevos temas o ideas durante las entrevistas. Después de la duodécima entrevista, se identificaron patrones recurrentes y dos entrevistas adicionales confirmaron la redundancia temática.

### Herramientas de Recolección de Datos

Las herramientas de recolección de datos consistieron en un formulario de información personal con datos introductorios sobre las participantes y un formulario de entrevista semiestructurada.

***Formulario de información personal:*** El formulario constaba de un total de cuatro preguntas sobre la edad, el género, el año de formación y el estado civil de las estudiantes.

***Formulario de entrevista semiestructurada:*** Este formulario se elaboró de acuerdo con el objetivo de la investigación y la bibliografía pertinente^(9)^. El estudio tenía como objetivo evaluar los sentimientos, pensamientos y observaciones de las estudiantes de enfermería con respecto a la violencia obstétrica.

Basándose en la teoría fundamentada constructivista y en el objetivo de la investigación, se creó un formulario de entrevista semiestructurada que constaba de siete preguntas abiertas (Tabla 1) para recolectar datos entre enero y febrero de 2023 utilizando las herramientas mencionadas anteriormente.Se utilizó la guía de entrevista semiestructurada para garantizar la coherencia de las preguntas para todos los participantes, con las preguntas específicas enumeradas en la Tabla 1.

**Tabla 1:** Formulario de entrevista semiestructurada – Nevsehir, Turquía, 2023.

1	¿Qué te recuerda el concepto de violencia obstétrica
2	¿Qué características incluirías en tu definición de violencia obstétrica?
3	¿Cómo te sentiste cuando presenciaste la violencia obstétrica?
4	¿Qué creencias, valores o principios crees que están asociados con la violencia obstétrica y por qué?
5	¿Qué consecuencias cree que tiene la violencia obstétrica en las pacientes (físicas, psicológicas y sexuales)?
6	¿Qué estrategias considera necesarias para evitar la violencia obstétrica (a nivel individual, sanitario e institucional)?
7	¿Hay algo más que quieras agregar?

Se informó a los estudiantes (60) que recibieron formación en enfermería obstétrica y ginecológica y completaron las prácticas en la sala de partos sobre el proceso de investigación y se les invitó a participar en el estudio. Veinte estudiantes se inscribieron voluntariamente en el estudio. Se invitó a los estudiantes a participar en entrevistas en días y horas específicos establecidos por los investigadores. Las entrevistas se llevaron a cabo hasta alcanzar la saturación de datos. Cuando los conceptos y expresiones que respondían a las preguntas del estudio comenzaron a repetirse (es decir, cuando se alcanzó la saturación), se dio por concluida la recolección de datos. En este estudio, las entrevistas con 14 participantes se grabaron en audio con una grabadora de voz. La recolección de datos se llevó a cabo en un entorno adecuado (la sala de estudio de los investigadores). Los participantes y los investigadores se colocaron de manera que pudieran verse durante la entrevista. El primer investigador realizó la entrevista, mientras que el segundo investigador tomó notas de observación. Las preguntas se formularon en el mismo orden y se dieron explicaciones adicionales cuando fue necesario. El investigador hizo preguntas como “¿Puede dar más detalles?”, “¿Puede dar más detalles sobre esto?”, “¿Qué opina sobre este tema?”. Todas las opiniones expresadas se evaluaron como datos cualitativos. Las entrevistas duraron entre 30 y 35 minutos de media.

### Análisis de Datos

Los datos se analizaron utilizando el análisis temático de van Manen^(10)^. Se llevó a cabo un análisis textual en tres etapas, que consistió en (1) lectura ingenua, (2) análisis estructural y (3) una comprensión integral que incluyó discusión^(10)^. En la etapa de lectura ingenua, se estimaron el alcance y los significados del texto de manera ingenua, sin ningún análisis formal. En esta etapa, se leyó el texto de la entrevista varias veces para obtener una impresión general. En la etapa de análisis estructural, se crearon códigos iniciales para descubrir los significados, y los primeros códigos se clasificaron en temas. Los temas fueron generados inductivamente por los investigadores de acuerdo con el análisis temático reflexivo. La codificación fue realizada por separado por dos investigadores, y cualquier inconsistencia entre los codificadores se discutió hasta llegar a un consenso. Antes de determinar los temas analíticos, se identificaron los códigos más destacados, sus descripciones y las relaciones entre ellos, y luego se combinaron en temas descriptivos mediante la síntesis temática. En la etapa final, los datos obtenidos en las dos etapas anteriores y los objetivos del estudio se revisaron en su conjunto a la luz de la bibliografía.

### Fiabilidad y Validez del Estudio

La validez y fiabilidad de este estudio se garantizaron mediante el rigor, la coherencia, la transferibilidad y la confirmabilidad. Antes de la entrevista, se proporcionó la información necesaria para garantizar una comunicación segura entre el participante y el investigador. Con el consentimiento del participante, un investigador realizó las entrevistas mientras el otro observaba. Durante las entrevistas, los investigadores evitaron las preguntas capciosas y, al final de la entrevista, se preguntó al participante si tenía algo que añadir y se registraron sus comentarios y opiniones adicionales. Las transcripciones de las entrevistas se compartieron con los participantes al final de la entrevista y se obtuvo su consentimiento informado. La validez del estudio se respaldó incluyendo con frecuencia declaraciones directas de los participantes en el manuscrito. Para garantizar la confirmabilidad, se utilizaron las notas de las entrevistas (grabaciones de voz) como datos brutos o se tomaron notas sobre las declaraciones de los participantes durante la entrevista, y sus declaraciones se incluyeron directamente en el informe de la investigación. Además, se explicó detalladamente el diseño del estudio (preguntas de investigación, recolección de datos y evaluación de datos) para garantizar la coherencia. De este modo, se garantizó que los lectores comprendieran el método y el estudio. Los investigadores creen que los resultados de las entrevistas de este grupo de muestra son transferibles y coherentes, ya que pueden aplicarse a grupos de muestra similares en diferentes entornos.

### Conocimiento Autorreflexivo de los Investigadores

El conocimiento autorreflexivo de los investigadores de este estudio es el siguiente: el primer y el segundo investigador completaron sus doctorados (PhD) en el campo de la obstetricia y la enfermería ginecológica. Los investigadores tienen experiencia en investigación científica en ginecología e investigación cualitativa. Además, la primera autora ha dado a luz una vez, una experiencia que, en cierta medida, le ha facilitado la comprensión de las experiencias de otras mujeres. Además, la primera autora tuvo un embarazo de alto riesgo y pasó 52 días en la sala de ginecología por riesgo de parto prematuro y recibió atención médica. Además, la experiencia de la investigadora como enfermera de ginecología garantizó tanto la comunicación eficaz con las participantes como la coherencia del proceso de entrevista.

### Aprobación Ética

Se obtuvo la aprobación por escrito del Comité de Ética de la universidad correspondiente antes de iniciar la investigación (fecha: 21.11.2022, n.º de referencia: 2022.10.108). Se obtuvo el permiso por escrito de la institución correspondiente para llevar a cabo la investigación (fecha: 09.12.2022, n.º de referencia: 51301242-200-2200001428). El estudio se llevó a cabo de acuerdo con los principios de la Declaración de Helsinki, y se obtuvo el consentimiento informado por escrito de cada participante, explicándole el propósito del estudio e indicándole que se utilizaría una grabadora de voz durante la entrevista. Los nombres de los participantes se mantuvieron confidenciales y se codificaron como P1, P2, …, P14 (en lugar de sus nombres completos).

## RESULTADOS

El 71,4% (n = 10) de los estudiantes eran mujeres y el 28,6% (n = 4) eran hombres. Sus edades oscilaban entre los 21 y los 31 años (21,50 ± 2,79). El análisis temático produjo dos estratos conceptuales en línea con el marco de nivel micro/macro propuesto por Mena-Tudela et al.^(9)^: **Los temas de nivel micro** incluyeron: (1) *Falta de información y consentimiento*, (2) *Acoso verbal* y (3) *Pérdida de valor profesional*, que abarcaba los subtemas Barrera a la educación y Modelos de conducta negativos. (4) *Falta de comunicación efectiva*, que comprendía los subtemas Barreras lingüísticas y Uso de lenguaje vulgar. El **tema de nivel macro** fue *Búsqueda de soluciones*, e incorporó los subtemas Concienciación y Empatía (Tabla 2).

**Tabla 2:** Temas y subtemas que surgieron de las entrevistas – Nevsehir, Turquía, 2023

Nivel del modelo conceptual		Temas	Subtemas	Declaraciones de los participantes
Estrategias a nivel micro	Cuidado no autorizado	Falta de información y consentimiento		Los estudiantes afirmaron que la información proporcionada a las mujeres durante el parto era incompleta e inadecuada y que, en algunos casos, no se les proporcionaba ninguna información.
	Abuso verbal e infantilización de las mujeres	Acoso verbal		Los estudiantes informaron que las parteras gritaban a las mujeres, las insultaban, hacían comentarios negativos y utilizaban expresiones despectivas.
	Jerarquía de relaciones en el sistema sanitario	Pérdida de valores profesionales	Una barrera para la educación	Los estudiantes afirmaron que observar la violencia obstétrica afectaba negativamente a su educación y que esta situación reducía su deseo de aprender.
			Modelo negativo a seguir	Los estudiantes afirmaron que el personal sanitario era un modelo negativo debido a su actitud hacia los pacientes y consideraron que esta situación perjudicaría el desarrollo de sus competencias.
	Embarazo y parto Intervención y medicalización innecesaria	Falta de comunicación efectiva	Barreras lingüísticas	El problema de comunicación dañó la relación entre la partera y la mujer, allanando el camino para prácticas negativas contra las mujeres y preparando el terreno para intervenciones que no deberían realizarse.
			Uso de lenguaje vulgar	Los estudiantes afirmaron que el lenguaje grosero utilizado por las parteras al comunicarse con las mujeres les impedía recibir servicios de salud.
Estrategias a nivel macro	Intervenciones institucionales, organizativas y políticas	En busca de soluciones	Concienciación	Los estudiantes afirmaron que se debería impartir formación continua para aumentar la concienciación de los profesionales de salud sobre la prestación de una atención humanitaria.
			Empatizar	Los estudiantes afirmaron que los trabajadores de salud no deben normalizar la violencia obstétrica y deben empatizar con la persona o con un familiar que pueda encontrarse en la misma situación. Algunos estudiantes afirmaron que se deben impartir cursos de formación periódicos para mejorar las habilidades de empatía de los profesionales de salud

### Estrategias a Nivel Micro

#### Tema 1. Falta de Información y Consentimiento

Los estudiantes afirmaron que la información proporcionada a las mujeres durante el parto era incompleta e inadecuada. En algunos casos, no se proporcionaba ninguna información a las mujeres, ya que, aunque solicitaban aclaraciones sobre las decisiones tomadas y las prácticas, no se les informaba. El consentimiento de las mujeres también se ignoraba durante los procedimientos. Los estudiantes informaron de que las parteras no dialogaban mucho con las mujeres, en particular con las mujeres extranjeras (en su mayoría refugiadas sirias), e ignoraban la mayoría de sus decisiones. Según muchos estudiantes, la mayoría de las mujeres embarazadas que acudían a la sala de partos estaban ansiosas porque no conocían el proceso, y las parteras no les daban ninguna explicación al respecto. Afirmaron que las parteras no tenían en cuenta la decisión de la mujer y que se le practicaba una cesárea sin ninguna explicación.

“*Fui testigo de que no proporcionaban ninguna información sobre cuándo sería dada de alta la paciente y cómo continuaría el tratamiento*”. (P8)

“*El bebé de la mujer fue trasladado a la unidad de cuidados intensivos neonatales después del parto. Sin embargo, ni el médico ni la enfermera informaron a los familiares de la paciente sobre el estado del bebé*”. (P5)

“*Las enfermeras proporcionaron cuidados y tratamiento a las pacientes en la sala, pero no les proporcionaron ninguna información ni explicación*”. (P8)

#### Tema 2. Acoso Verbal

Los estudiantes informaron de que las parteras gritaban a las mujeres, las insultaban, hacían comentarios negativos y utilizaban expresiones despectivas. A veces, este trato a las mujeres también estaba relacionado con su decisión de concebir o su futura capacidad para dar a luz. Las parteras a veces amenazaban a las mujeres con no prestarles asistencia durante el parto si no empujaban con eficacia.

Estas experiencias contrastaban profundamente con los modelos ideales de comunicación terapéutica que se enseñan en la formación de enfermería.

“*En realidad, lo que nos enseñan y el entorno real de la sala de partos son muy diferentes. Se suponía que debíamos proporcionar apoyo psicológico a la mujer. Probablemente, nuestro apoyo psicológico habría reducido su dolor. Sin embargo, las parteras se comportaban de manera opuesta, de una forma que aumentaba el dolor de la mujer o la traumatizaba*”. (P12)

“*Recuerdo a una partera gritándole a una mujer: ‘Llevas cuatro días aquí, nos has estado molestando, ¿no se supone que debes dar a luz? Da a luz ya, basta es basta’*”. (P3)

“*La mujer estaba pujando, tenía dolor, y las parteras y enfermeras no dejaban de gritarle, burlarse de ella y decirle cosas insultantes como: “¿Cómo diste a luz antes? ¿Cómo vas a dar a luz? ¿De verdad diste a luz a dos hijos? No vuelvas a dar a luz*”. (P7)

#### Tema 3: Falta de Comunicación Eficaz

##### Subtema 1: Barreras Lingüísticas

Los estudiantes afirmaron que la barrera lingüística provocaba dificultades de comunicación entre las mujeres extranjeras y las parteras. La mayoría de las mujeres extranjeras eran de nacionalidad siria y su lengua materna era el árabe. El hecho de que ninguna de las parteras hablara árabe era un obstáculo importante para una comunicación eficaz. Por lo tanto, este problema de comunicación dañaba la relación entre la partera y la mujer, allanando el camino para prácticas negativas contra las mujeres y preparando el terreno para intervenciones que no deberían realizarse.

“*Las extranjeras dan a luz con frecuencia y tienen problemas de idioma, y creo que los trabajadores sanitarios tienen dificultades para prestar asistencia porque no entienden el idioma hablado*”. (P7)

Estas fallas en la comunicación afectan el estado físico y emocional de las mujeres en trabajo de parto.

“*Las mujeres pasan hambre durante largos períodos de tiempo durante el parto y las parteras no pueden explicarles las razones a las mujeres extranjeras. Esto afecta la capacidad de las mujeres para concentrarse en el parto*”. (P6)

“*Las parteras realizaban exámenes vaginales todo el tiempo. Algunas mujeres incluso cerraban las piernas y se negaban a que se los hicieran*”. (P1)

##### Subtema 2: Uso de Lenguaje Vulgar

Los estudiantes destacaron que les molestaba el enfoque de las parteras al comunicarse con las mujeres. Las parteras a menudo se dirigían a las mujeres con impaciencia y falta de comprensión, utilizando imperativos como “haz esto, haz aquello”, lo que puede hacer que las mujeres se retraigan y no reciban la atención médica necesaria.

Este tono de mando alejaba aún más a las pacientes de hacer preguntas o comprender su atención.

“*Cuando la paciente pregunta algo, a menudo le responden con dureza, lo que hace que la paciente se retraiga aún más y no sea capaz de preguntar sobre las prácticas que le interesan y sobre las que quiere informarse*”. (P2)

#### TEMA 4: Pérdida de Valor Profesional

Los estudiantes informaron de que la violencia obstétrica que observaban les afectaba negativamente y que esta situación era un ejemplo negativo para su profesión y constituía un obstáculo para su educación.

##### Subtema 1: Obstáculo para la Formación

Los estudiantes afirmaron que observar la violencia obstétrica afectaba negativamente a su formación y que esta situación reducía su deseo de aprender.

La exposición a la violencia en entornos clínicos distraía a los estudiantes de los objetivos formativos.

“*Me centré más en la violencia cuando debería estar observando el parto vaginal que se explicaba en clase*”. (P2)

“*Era la primera vez que veía un parto. Pensé que era algo muy doloroso. Pensé que era humillante... Para mí fue un caos y me sentí más estresada*”. (P7)

Los estudiantes expresaron su dificultad para conciliar los conocimientos teóricos con la realidad clínica.

“*No es así como nos lo enseñaron; en el momento en que intentamos hacerlo como nos enseñaron, sentimos que no podemos comunicarnos eficazmente con la paciente*”. (P10)

##### Subtema 2: Modelo Negativo

Los estudiantes afirmaron que el personal sanitario era un modelo negativo debido a su actitud hacia los pacientes y pensaban que esta situación perjudicaría el desarrollo de sus competencias. De hecho, los estudiantes observan a sus mentores, los aceptan como modelos a seguir y aprenden de ellos. Este modelo puede hacer que interioricen los valores profesionales de forma negativa. Es fundamental preparar a la próxima generación de enfermeros para un entorno de trabajo profesional. Las actitudes negativas hacia las mujeres pueden amenazar el progreso y la motivación de los estudiantes.

“*En nuestros cuatro años de formación universitaria, siempre hemos aprendido métodos de comunicación terapéutica y a ser comprensivos con los pacientes. Ser testigo de actitudes tan negativas en el hospital mientras aprendo estas cosas me hace preocuparme por si yo seré así en el futuro*”. (P13)

Otro estudiante afirmó claramente cómo esta discrepancia entre la teoría y la práctica le llevó a un retraimiento emocional:

“*No era lo que había aprendido. Me desmotivó mucho*”. (P12)

El costo emocional de presenciar la violencia obstétrica iba más allá de la desmotivación. Algunos estudiantes se sintieron profundamente traumatizados, aislados y abandonados, y expresaron experiencias de profunda impotencia:

“*Estás sola, vas a dar a luz y te sientes muy sola. La persona más cercana a la que una paciente puede aferrarse en un hospital es un trabajador sanitario, pero no recibes apoyo. Fue un acontecimiento muy traumático para mí y me afectó mucho*” (P3).

Otro participante describió un impacto emocional similar al presenciar un trato irrespetuoso:

“*Era la primera vez que veía un parto. Pensé que era algo muy doloroso. Me pareció humillante y me sentí muy molesto. Hay que encontrar una solución a esta situación y, si es necesario, desarrollar estrategias adecuadas. Si la razón de esto es la falta de personal, hay que aumentar el número de empleados*” (P7).

En general, estas narrativas demuestran que los modelos de conducta negativos del personal sanitario pueden afectar profundamente a los estudiantes, provocándoles un importante malestar emocional, sentimientos de desesperanza y comprometiendo potencialmente su motivación, orientación ética y preparación profesional. Garantizar que los estudiantes cuenten con mentores compasivos y con una base ética es esencial para fomentar que los futuros profesionales de la salud se comprometan con una atención respetuosa, empática y humana.

### Estrategias a Nivel Macro

#### Tema 5. Búsqueda de Soluciones

Los estudiantes afirmaron que la solución a la violencia obstétrica es aumentar la concienciación del personal de atención de salud y empatizar con ellos.

##### Subtema 1: Concienciación

Los estudiantes recomendaron que la formación continua de los profesionales de atención de salud se centrara en mejorar la comunicación eficaz y proporcionar información detallada sobre el proceso y los procedimientos del parto para apoyar a las mujeres durante el parto, aumentando así la concienciación sobre la necesidad de proporcionar una atención humana y solidaria a las mujeres. La mayoría de los estudiantes afirmaron que la información sobre la violencia obstétrica debería impartirse en su formación universitaria para sensibilizar a sus compañeros.

“*Creo que se puede formar a las enfermeras en materia de empatía y mejorar sus habilidades empáticas*.” (P8)

Algunos también abogaron por la integración temprana de temas relacionados con la violencia obstétrica en la formación oficial de enfermería.

“*Nuestros profesores hablan constantemente de lo que debemos hacer de la A a la Z y de cómo debemos abordar al paciente. Proporcionar información sobre la violencia obstétrica en nuestras clases aumentará nuestra conciencia sobre este tema. Esto evitará que, sin saberlo, dañemos a la paciente en el futuro. Creo que es necesaria una formación integral*”. (P2)

##### Subtema 2: Empatía

Los estudiantes afirmaron que los trabajadores sanitarios no deben normalizar la violencia obstétrica y deben empatizar con la persona o el familiar que pueda encontrarse en la misma situación. Algunos estudiantes afirmaron que se deben impartir capacitaciones periódicas para mejorar las habilidades de empatía de los profesionales de la salud. Estos comentarios subrayaron la importancia de la práctica reflexiva y los valores humanísticos en la atención clínica.

“*Deben brindar atención preguntándose: Si yo estuviera en esta situación, ¿cómo querría que me trataran? Y nunca deben normalizar la violencia*”. (P14)

“*Los trabajadores de la salud nunca deben olvidar que la persona que tienen delante es un ser humano*”. (P12)

“*Es muy importante adoptar un enfoque consciente y empático. Como mujeres, todas podemos encontrarnos en esta situación en cualquier momento. No hago a otra persona algo que no me gustaría que me hicieran a mí, ni hago nada que pueda dar lugar a una queja; creo que eso no es un comportamiento ético en absoluto. Además, para mejorar las habilidades empáticas de los trabajadores sanitarios, se les debería impartir formación periódica y recordarles esta situació*n”. (P6)

## DISCUSIÓN

En este estudio, se examinaron las experiencias de las estudiantes de enfermería relacionadas con la violencia obstétrica utilizando un diseño exploratorio cualitativo y un análisis temático reflexivo. Los temas que surgieron del estudio indican violencia psicológica y verbal en las narrativas y observaciones de las estudiantes de enfermería. Las estudiantes informaron que las mujeres se veían expuestas con frecuencia a formas de violencia verbal, como insultos, gritos, amenazas y regaños durante el parto. Del mismo modo, muchos estudios encontraron que las mujeres se enfrentan a actitudes negativas, irrespetuosas y deshonrosas durante el parto^(11,12)^. Un estudio informó que las mujeres estaban expuestas a comportamientos negativos, incluyendo abusos físicos como bofetadas y estigmatización, discriminación como encerrarlas en el baño, y abusos verbales como gritos e intimidación^(12)^. En un estudio que examinaba las experiencias de parto de las mujeres que dieron a luz en centros de salud de países de ingresos bajos y medios, las mujeres se veían sometidas con frecuencia a experiencias de parto negativas, como negligencia, abandono, denegación de analgesia, falta de consentimiento informado y exámenes vaginales dolorosos^(11)^. En algunos casos, los trabajadores sanitarios pueden no ser conscientes de la violencia obstétrica y abusar de su autoridad profesional para socavar los derechos, la libertad, la capacidad de decisión y, en última instancia, la autonomía de las mujeres^(13)^. De hecho, en el presente estudio, los estudiantes informaron de que las mujeres no participaban en las decisiones y las intervenciones, y que los trabajadores sanitarios a menudo no daban ninguna explicación. De manera similar, otro estudio informó que se realizaron intervenciones médicas sin el consentimiento o permiso de las mujeres durante el parto y el alumbramiento, y que se las engañó o no se les informó suficientemente^(14)^. Según la Carta Europea de los Derechos de los Pacientes^(15)^, “toda persona tiene derecho a acceder a la información sobre su estado de salud, los servicios de salud y cómo utilizarlos, así como a la información generada por la investigación científica y la innovación tecnológica”. Por lo tanto, todas las parteras/enfermeras deben informar a las mujeres antes de cada procedimiento, teniendo en cuenta la autonomía de las mujeres.

La enfermería culturalmente competente se define como la atención sensible a las diferentes características culturales, como el idioma, la comunicación, las creencias y el comportamiento, que en última instancia puede mejorar los resultados de la atención médica^(16)^. Entre estas características, la comunicación reviste especial importancia, ya que la existencia de un idioma común es un requisito básico para mantener la atención y evaluar su eficacia^(17)^. Muchos estudios han demostrado que las barreras comunicativas y lingüísticas se encuentran entre los problemas más comunes a la hora de prestar atención a pacientes extranjeros^(17,18)^. Una observación importante de las estudiantes de enfermería que participaron en el presente estudio fue la barrera lingüística. Según ellas, existía un problema de comunicación entre las mujeres extranjeras y las parteras debido a las barreras lingüísticas, lo que allanaba el camino para prácticas negativas hacia las mujeres. Para superar esta situación, contar con un número suficiente de intérpretes que ayuden a las enfermeras a comunicarse con las pacientes extranjeras en las instituciones de salud sería un paso importante para garantizar una atención digna durante el parto.

La violencia en la atención materna hacia las mujeres puede generar desconfianza en el sistema sanitario y en las estructuras de apoyo existentes. Un estudio mostró que las mujeres describían el hospital y los profesionales de la salud como un entorno odioso^(19)^. Otro estudio informó de que la desconfianza de las madres en el sistema de atención de salud debido a la violencia obstétrica es un fuerte factor disuasorio y afecta negativamente a sus futuros comportamientos de búsqueda de atención médica^(20)^. Las tareas más importantes de las enfermeras son eliminar esta situación, garantizar que las mujeres reciban la mejor atención posible y prestar servicios que defiendan la dignidad humana. En el presente estudio, los estudiantes sugirieron aumentar la concienciación de las enfermeras como solución a la violencia obstétrica. Otro estudio mostró la posibilidad de cambiar esta percepción con la implementación de la educación sobre la violencia obstétrica^(21)^. Argumentaron que las políticas, las directrices, los protocolos y la educación, así como la formación que promueve el respeto, informa y sensibiliza a los profesionales de la salud, eliminarán la violencia obstétrica. Además, proporcionar información sobre el parto a todas las mujeres en el periodo prenatal puede mejorar la experiencia del parto.

Además del impacto negativo de la violencia obstétrica en la vida de las mujeres, los testigos pueden experimentar estrés traumático secundario o fatiga por compasión^(22)^. Los resultados obtenidos en el presente estudio son coherentes con la literatura, y los estudiantes informaron que presenciar la violencia obstétrica les hacía sentir mal. Por otra parte, la práctica clínica en la formación de enfermería constituye un componente esencial del proceso educativo, ya que ofrece a los estudiantes la oportunidad de aplicar los conocimientos teóricos a situaciones prácticas^(23)^. El estudiante puede verse influido, ya sea positiva o negativamente, por las habilidades, los conocimientos y las actitudes que demuestran las enfermeras profesionales en el entorno clínico. Los modelos de conducta positivos mejoran significativamente las habilidades de cuidado de los estudiantes de enfermería en la práctica clínica. En particular, las habilidades de enfermería de los estudiantes de enfermería se ven influenciadas por el comportamiento humano y las habilidades de comunicación respetuosa que observan en sus modelos a seguir^(24)^. Ser testigo de prácticas inapropiadas y tener modelos a seguir negativos afecta negativamente al desarrollo de la identidad profesional de los estudiantes de enfermería^(25)^. De hecho, en este estudio, los estudiantes de enfermería informaron que las parteras que participaban en violencia obstétrica eran percibidas como un modelo a seguir negativo, lo que afectaba negativamente a su educación y disminuía su deseo de aprender. De manera similar a estos hallazgos, investigaciones anteriores indican que la exposición de los estudiantes a modelos a seguir negativos en el entorno clínico afectaba negativamente a su aprendizaje y daba lugar a sentimientos negativos hacia la profesión de enfermería^(26)^. Además, la exposición de los estudiantes de enfermería a comportamientos poco éticos mostrados por enfermeros profesionales hacia los pacientes puede llevarles a normalizar y adoptar dichos comportamientos. Los estudiantes de enfermería, que se encuentran en los inicios de su profesión, deben tener modelos a seguir competentes que den prioridad a la salud a largo plazo de la sociedad. Esto garantiza que puedan prestar una atención eficaz y satisfactoria a los pacientes, al tiempo que proporcionan una atención materna digna en las clínicas obstétricas. Además, la incorporación de la violencia obstétrica en los planes de estudios de enfermería puede aumentar la concienciación de las enfermeras sobre esta cuestión y puede ser un paso importante para prevenir la violencia obstétrica.

### Limitaciones

A pesar de los esfuerzos por maximizar la diversidad de la muestra, los resultados de este estudio cualitativo, debido a su naturaleza inherente, no pueden generalizarse a todas las estudiantes de enfermería de Turquía. Los resultados obtenidos en el presente estudio se limitan al análisis de datos cualitativos y al entorno cultural y la situación socioeconómica de las estudiantes de enfermería que participaron en el estudio. Aunque los resultados no pueden generalizarse, las conclusiones del estudio son instructivas para otras investigaciones, ya que presentan detalles y categorías importantes como modelo conceptual e ilustran experiencias ejemplares.

### Repercusión en la Práctica y la Investigación

Para mejorar la calidad de los servicios de salud materna, las enfermeras deben desempeñar un papel activo en la creación de leyes que prevengan la violencia obstétrica, una clara violación de los derechos humanos de las mujeres. Para eliminar el maltrato de las mujeres en los servicios de salud materna, las regulaciones legales deben ir acompañadas de la formación de los profesionales de la salud en materia de ética, igualdad de género y derechos humanos, sensibilizándolos sobre estas cuestiones fundamentales. Para garantizar una transformación positiva en los servicios de salud materna, se deben realizar investigaciones utilizando diversas metodologías para explorar las causas, la prevalencia y las consecuencias de la violencia obstétrica. Al mismo tiempo, se deben desarrollar programas de formación eficaces.

## CONCLUSIÓN

Las estudiantes de enfermería que presenciaron violencia obstétrica durante su formación se vieron afectadas negativamente por esta situación y este suceso fue traumático para ellas. El maltrato a las mujeres durante el parto es un problema relacionado con la calidad de la atención y una violación de los derechos humanos. Todas las mujeres, independientemente del lugar del mundo en el que se encuentren, tienen derecho a recibir una atención sanitaria digna y libre de cualquier forma de abuso. Las enfermeras desempeñan un papel fundamental a la hora de garantizar que las mujeres reciban una atención materna de calidad durante el embarazo, el parto y el posparto. Se encuentran en una posición ideal para defender y proteger a las mujeres, ya que constituyen la primera línea de defensa contra una atención abusiva e irrespetuosa. Por último, se considera que el tema tiene sus méritos y que se debe valorar la comprensión y la conciencia temprana de los estudiantes, casi profesionales de la enfermería, hacia las prácticas que descuidan la buena atención y violan los derechos humanos y la autonomía de las mujeres embarazadas. Por lo tanto, los resultados de este estudio son valiosos para desarrollar estrategias de intervención que aborden la violencia obstétrica que sufren las mujeres y para prestar una atención de calidad, igualitaria y personalizada.

## Data Availability

Los datos que respaldan los resultados de este estudio están disponibles a través del autor correspondiente, previa solicitud razonable.
